# Supplementary material for: Piezo1 Regulation Involves Lipid Domains and the Cytoskeleton and Is Favored by the Stomatocyte–Discocyte–Echinocyte Transformation
Source: Biomolecules. 2023 Dec 30;14(1):51. doi: 10.3390/biom14010051 (PMC10813235; doi:10.3390/biom14010051)
Supplement: Supplementary file 1 [file biomolecules-14-00051-s001.zip › biomolecules-2748919-supplementary.pdf]

Supplemental Materials

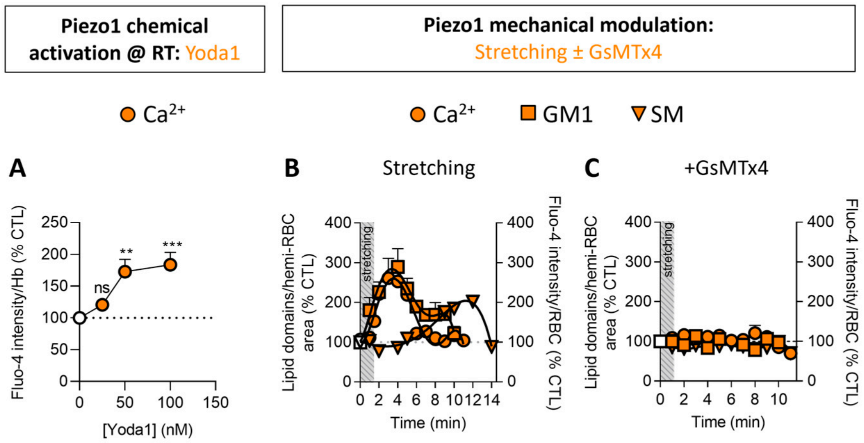

Figure S1. Piezo1 mechanical and chemical activation induces a calcium influx. Upon mechanical stimulation, this calcium influx is accompanied by a concomitant increase of GM1-enriched domains followed by a rise of SM-enriched domains, all abrogated by mechanosensitive channel inhibition. RBCs from healthy donors were analyzed for Ca^{2+} content upon chemical activation by Yoda1 **(A)** or mechanical activation in PDMS chambers **(B, C)** and lipid domains upon mechanical activation combined or not (B) with inhibition (C). White symbols, controls; orange, stretched- or chemically-activated RBCs. Data are from [47].

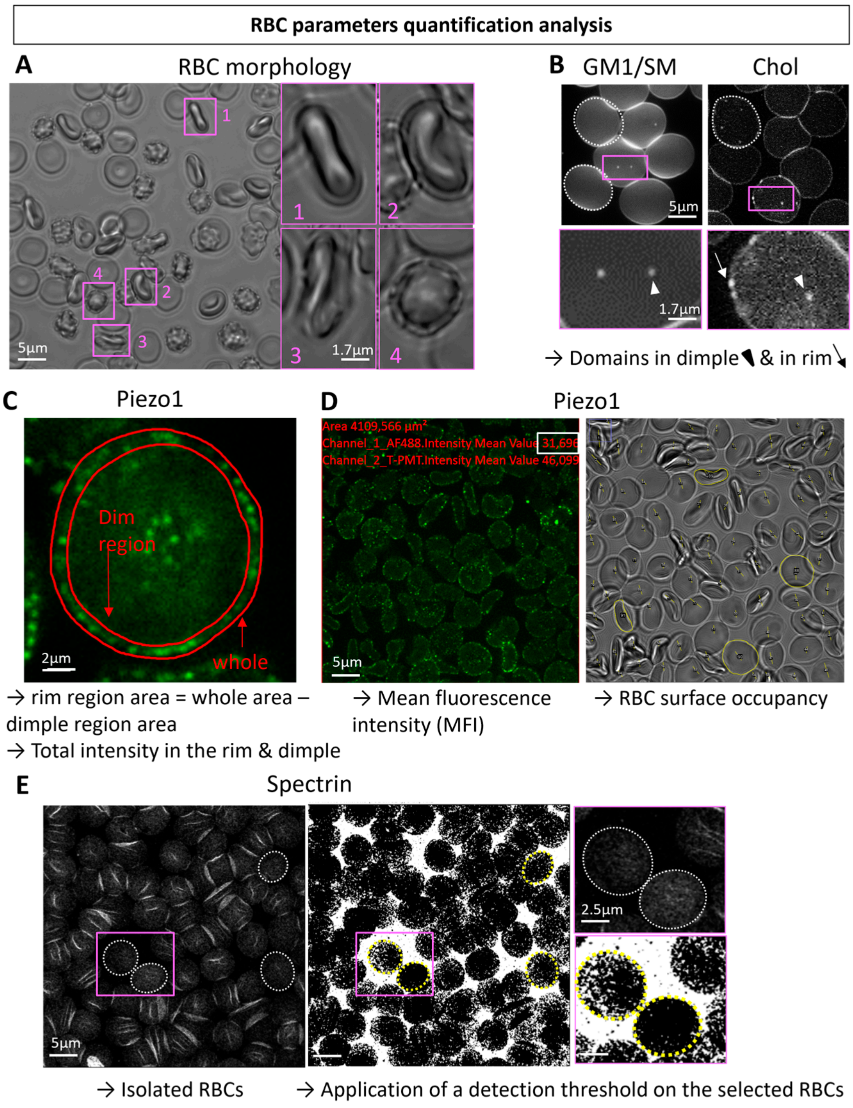

Figure S2. Schematic representation of the procedures used to quantify RBC morphology, lipid domains, Piezo1 and spectrin cytoskeleton. After chemical treatment, RBCs were visualized and quantified for the global morphology (A), lipid domains (B), Piezo1 fluorescence (C, D) and spectrin occupancy (E). **(A)** Morphology of RBCs in suspension determined in plastic IBIDI chambers and quantified for the proportion of each population per total RBC number. 1, discocytes; 2, stomatocytes; 3, echinocytes; 4, spherocytes. **(B)** Fluorescence imaging of RBCs spread on PLL and labeled with BODIPY-GM1 or BODIPY-SM or of RBCs decorated with mCherry-Theta toxin fragment as in Figure 1B and 4F. Data extracted are the number of lipid domains per hemi-RBC as well as the projected area size (white dotted line around RBC). **(C, D)** Confocal imaging of fixed and permeabilized RBCs spread on PLL and immunolabeled for Piezo1. Data extracted are the total fluorescence in the rim and the dimple regions and their respective area size (C) and the Piezo1 MFI (D). **(E)** Confocal imaging of RBCs spread on PLL, permeabilized, fixed and immunolabeled for spectrin. Data extracted is the importance of the membrane:cytoskeleton anchorage via the spectrin occupancy.

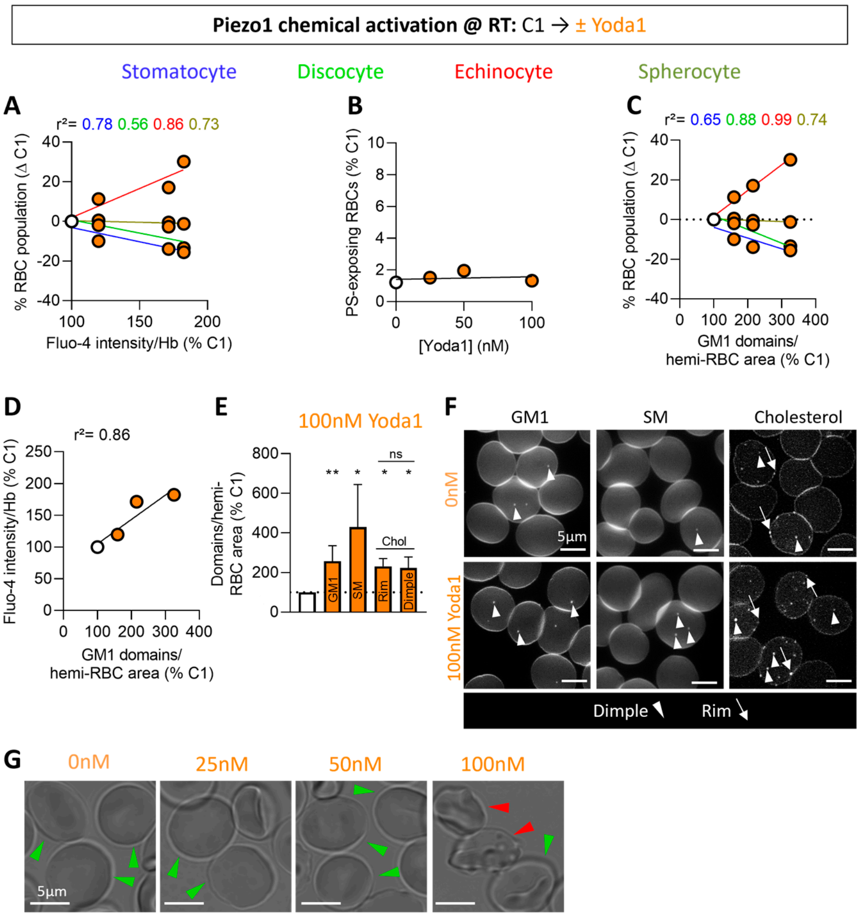

Figure S3. Piezo1 chemical activation by Yoda1 increases the abundance of all types of lipid domains but preserves the RBC transversal asymmetry. RBCs from one healthy donor (C1) were incubated in DMEM at RT for 20min in suspension and then with the Yoda1 for 30sec (white, controls; orange, Yoda1) and analyzed for phosphatidylserine (PS) surface exposure (B), lipid domains (E,F) and morphology after fixation (G) or parameters were correlated (A, C, D). **(A)** Correlation between RBC morphology proportions and the intracellular Ca^{2+} levels. Stomatocytes, blue; discocytes, green; echinocytes, red; spherocytes, brown-yellow. **(B)** PS exposure at the RBC surface determined by flow cytometry upon labeling with fluorescent Annexin-V. One experiment. **(C)** Correlation between RBC proportions and the GM1-enriched domain abundance. **(D)** Correlation between the intracellular Ca^{2+} levels and the GM1-enriched domain abundance. **(E, F)** Fluorescent imaging of RBCs labeled with BODIPY-GM1 or BODIPY-SM or endogenous chol decoration by the mCherry-Theta toxin fragment. (F) Representative images and (E) quantification of lipid domains as in Figure 1B, G and of chol-enriched domains in the rim and the dimple regions as in Figure 4F. White arrowheads, lipid domains in the dimple region; white arrows, lipid domains in the rim region. Mean ± SEM of 4-5 independent experiments where 100-300 RBCs per image were analyzed. **(G)** Morphology of RBCs fixed in suspension and spread on PLL-coated coverslips. Discocytes, green; echinocytes, red. T-test, Kruskal-Wallis with Dunn's multiple comparison and Mann-Whitney test.

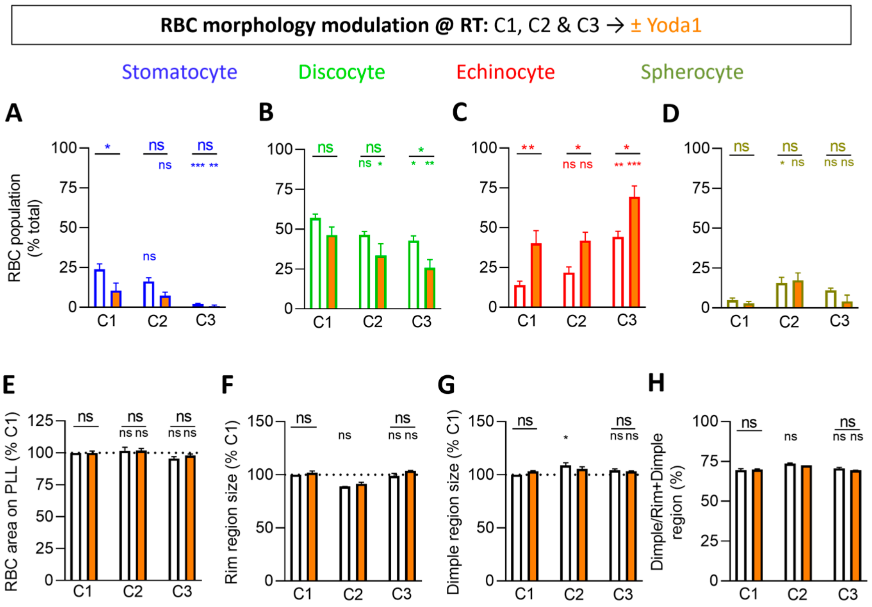

Figure S4. The differential RBC morphology of the three healthy donors included in this study does not result from differential RBC total surface or rim and dimple region areas. RBCs from three healthy donors (C1-C3) were incubated at RT for 20min in suspension and then with Yoda1 for 30sec (white, controls; orange, Yoda1). After Yoda1 incubation, RBCs were analyzed for the RBC morphology (A-D), RBC projected area on PLL (E), size of the local rim region (F) and dimple region (G) and proportion of the dimple region over the rim one (H). **(A-D)** Quantification of the proportions of each RBC population per total RBC number determined as in Figure 1C-F. Stomatocytes, blue; discocytes, green; echinocytes, red; spherocytes, brown-yellow. Mean ± SEM of 3-5 independent experiments where 50-250 RBCs per condition were manually counted. **(E-H)** Area size determination of living RBCs spread on PLL (E) and of fixed RBCs spread on PLL (F-H). (E) Mean ± SEM of 4-5 independent experiments where 20 RBCs per image were analyzed. (F-H) Mean ± SEM of 3 independent experiments (except for C2+Yoda1, mean ± SD of 2) where 19-31 RBCs were analyzed. Kruskal-Wallis with Dunn's multiple comparison and Mann-Whitney test.

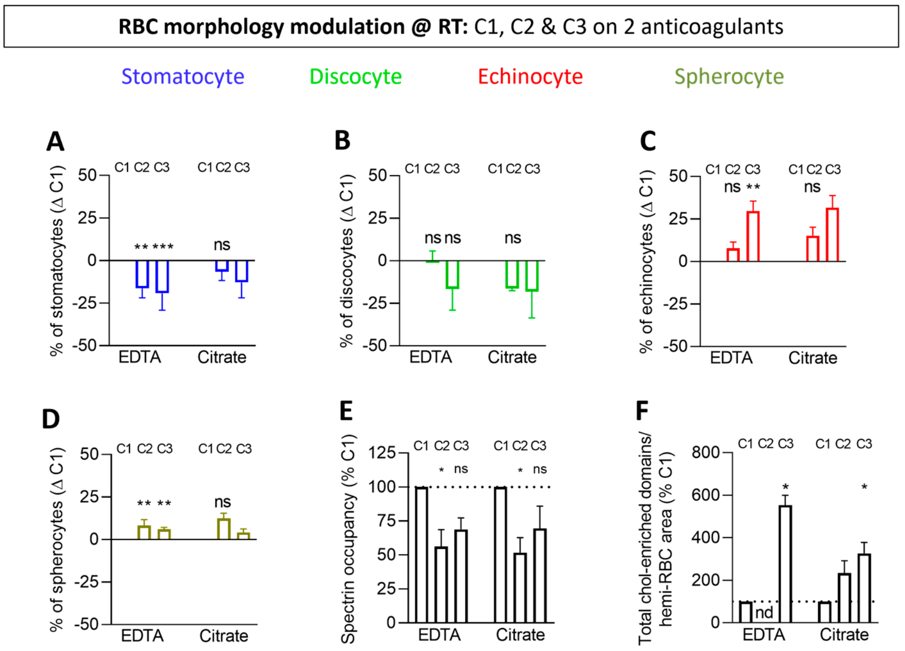

Figure S5. The differential RBC morphology of the three healthy donors included in this study does not result from the anticoagulant used for venipuncture but can be associated with differential spectrin membrane occupancy and chol-enriched domain abundance. RBCs from three healthy donors (C1-C3) were collected into K^+/EDTA- or citrate-coated tubes. After 20min at RT, RBCs were analyzed for RBC morphology (A-D), the spectrin membrane occupancy (E) and the chol-enriched domains (F). **(A-D)** Quantification of the proportions of each RBC population per total RBC number determined as in Figure 1C-F. Stomatocytes, blue; discocytes, green; echinocytes, red; spherocytes, brown-yellow. Mean ± SEM of 3-8 independent experiments (except for C3-citrate, mean ± SD of 2) where 50-250 RBCs (EDTA) or 100-170 RBCs (citrate) per condition were analyzed. **(E)** Quantification of spectrin membrane occupancy determined as in Figure 3H. Mean ± SEM of 4-5 independent experiments where 28-68 RBCs (EDTA) or 45-63 RBCs (citrate) per condition were analyzed. **(F)** Quantification of chol-enriched domains in the rim and the dimple regions as in Figure 4F. Mean ± SEM of 4 (except for C2 citrate Mean ± SD of 2) independent experiments where 70-125 RBCs per fluorescent images were analyzed. Kruskal-Wallis with Dunn's multiple comparison and Mann-Whitney test.

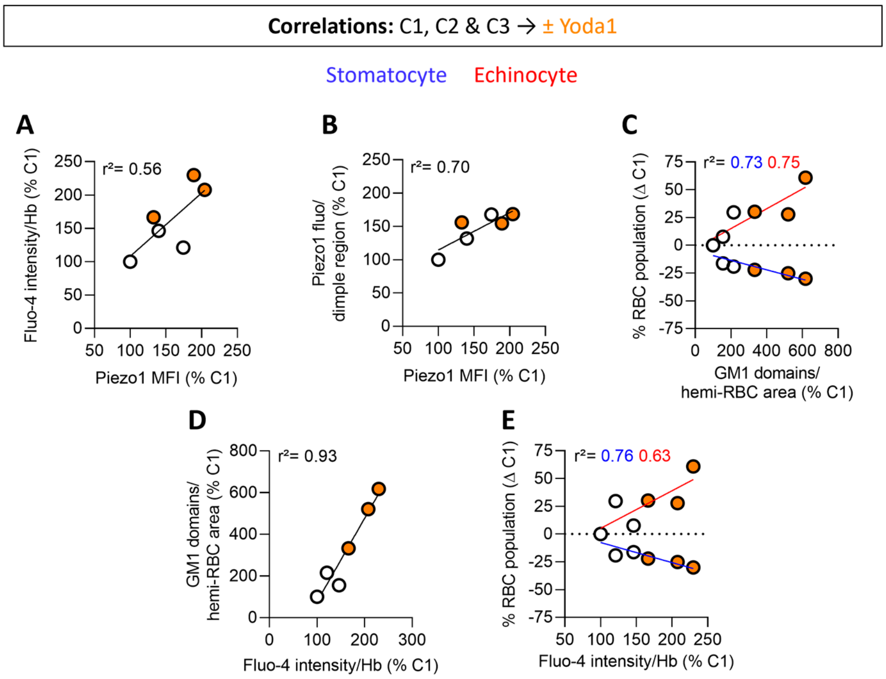

Figure S6. Correlations between RBC morphology, calcium content, GM1-enriched domain abundance and Piezo1 parameters. RBCs from three healthy donors (C1-C3) were incubated at RT for 20min in suspension and then with Yoda1 for 30sec (white, controls; orange, Yoda1-treated RBCs). **(A)** Correlation between the intracellular Ca^{2+} levels and the Piezo1 MFI. **(B)** Correlation between the Piezo1 fluorescence in the dimple region and the Piezo1 MFI. **(C)** Correlation between the RBCs morphology proportions and the GM1-enriched domain abundance. **(D)** Correlation between the GM1-enriched domain abundance and the intracellular Ca^{2+} levels. **(E)** Correlation between the RBC morphology proportions and the intracellular Ca^{2+} levels.

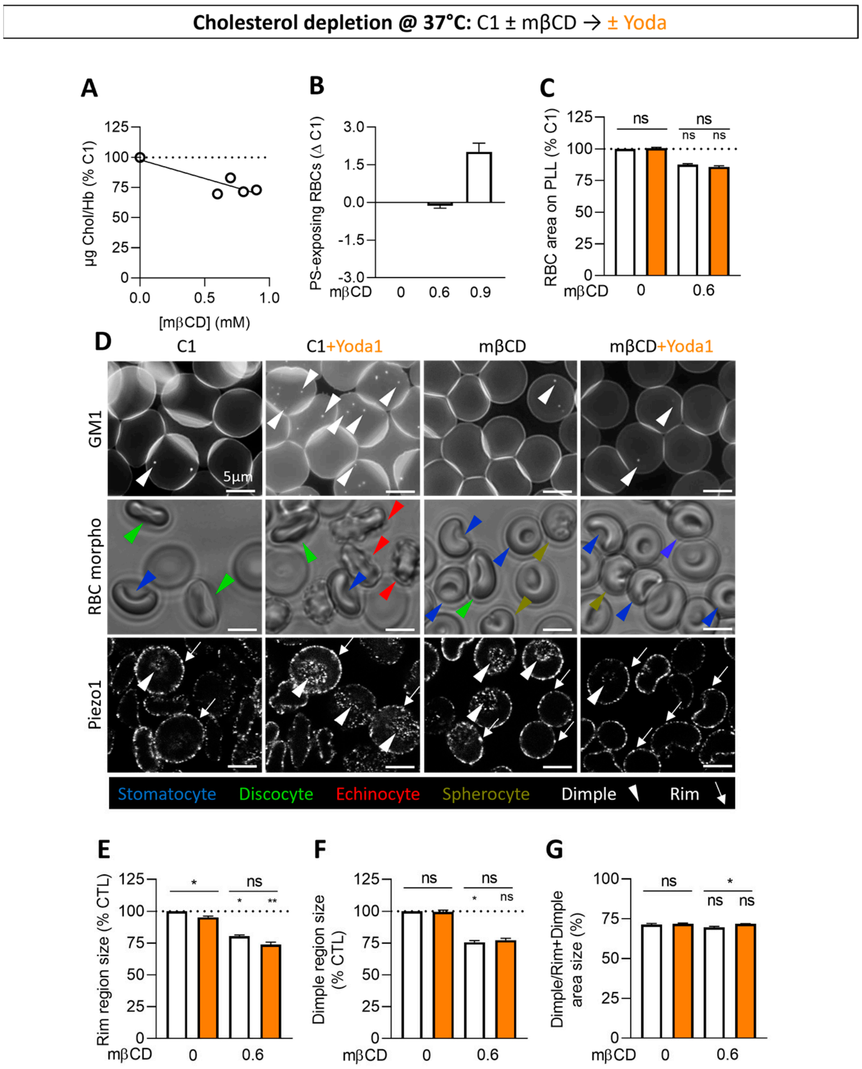

Figure S7. Cholesterol depletion by 0.6mM methyl-β-cyclodextrin induces partial RBC surface area loss but does not affect membrane transversal asymmetry in contrast to 0.9mM. RBCs from one healthy donor (C1) were incubated at 37°C with mβCD for 15min and then with Yoda1 for 30sec (white, controls; orange, Yoda1-treated RBCs). After chol depletion, RBCs were analyzed for the membrane chol content (A), PS surface exposure (B), RBC projected area on PLL (C), GM1-enriched domains, RBC morphology proportions and Piezo1 fluorescence distribution (D), size of the local rim region (E) and dimple region (F) and proportion of the dimple region area over the rim one (G). **(A)** Membrane chol content determined by fluorimetry and normalized to Hb content. **(B)** PS surface exposure determined by labeling with fluorescent Annexin-V by flow cytometry. Triplicates ± SD of one experiment. **(C)** Area size determination of living RBCs as in Figure S4E. Mean ± SEM of 3-5 independent experiments where 20 RBCs per image were analyzed. **(D)** Representative images of data from Figure 5. 1st row: RBCs spread on PLL and labeled with BODIPY-GM1; white arrowheads, GM1-enriched domains in the dimple region. 2nd row: In suspension RBCs determined for morphology in plastic IBIDI chambers; stomatocytes, blue; discocytes, green; echinocytes, red; spherocytes, brown-yellow. 3rd row: Fixed and permeabilized RBCs spread on PLL and immunolabeled for Piezo1; white arrowheads, Piezo1 clusters in the dimple region; white arrows, Piezo1 clusters in the rim region. **(E-G)** Area size determination of fixed BCs as in Figure S4F-H. Mean ± SEM of 4 independent experiments where 23-55 RBCs per confocal images were analyzed. Kruskal-Wallis with Dunn's multiple comparison and Mann-Whitney test.

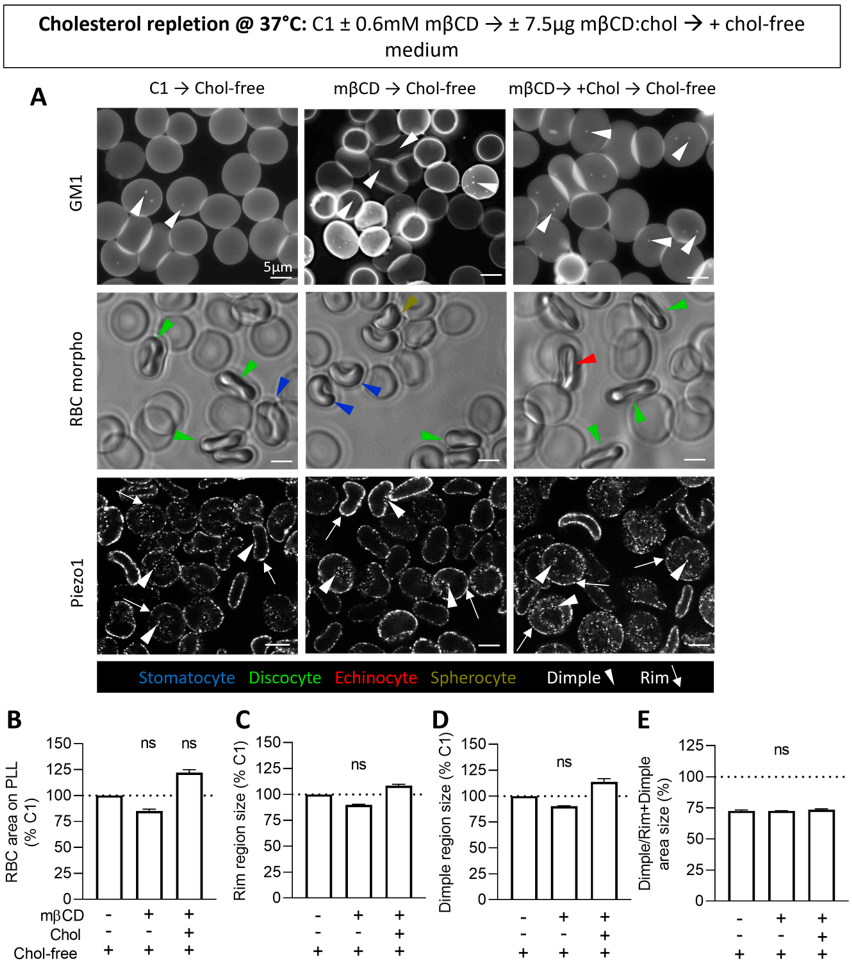

Figure S8. Cholesterol repletion restores and even increases the RBC surface area. RBCs from one healthy donor (C1) were either left untreated (1st column) or incubated with mβCD for 15min followed or not (2nd column) by treatment with mβCD:chol for 60min (3rd column). All RBCs were then reincubated in a chol-free medium for 90min. After chol depletion and/or repletion, RBCs were analyzed for GM1-enriched domains, RBC morphology proportions and Piezo1 fluorescence distribution (A), the RBCs projected area on PLL (B), size of the local rim region (C) and dimple region (D) areas and size of the dimple region area proportion over the rim one (E). **(A)** Representative images of data from Figure 6. 1st row: White arrowheads, GM1-enriched domains in the dimple region. 2nd row: Stomatocytes, blue; discocytes, green; echinocytes, red; spherocytes, brown-yellow. 3rd row: White arrowheads, Piezo1 clusters in the dimple region; white arrows, Piezo1 clusters in the rim region. **(B-E)** Area size determination of living (B) and fixed and permeabilized (C-E) as in Figure S4E-H. Mean ± SEM of 3 independent experiments (except for repleted condition mean ± SD of 2 in C-E) where 20 RBCs per fluorescent images were analyzed (B) and where 4-24 RBCs (C-E) per confocal images were analyzed. Kruskal-Wallis with Dunn's multiple comparison and Mann-Whitney test.

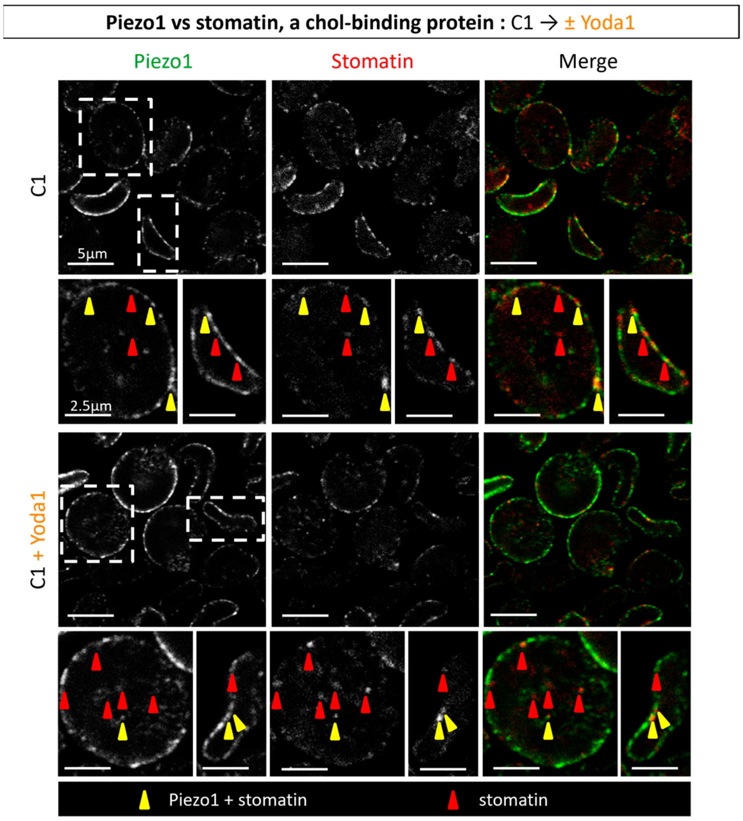

Figure S9. Piezo1 partly colocalizes with the cholesterol-binding protein stomatin particularly in the rim region of resting RBCs. RBCs from one healthy donor (C1) were incubated at RT for 20min in suspension and then with Yoda1 for 30sec. RBCs were then fixed in suspension, permeabilized, immunolabeled and analyzed for Piezo1 and stomatin distribution using confocal microscopy. Left, Piezo1; center, stomatin; right, merge. Yellow arrowheads, colocalization; red arrowheads, stomatin.

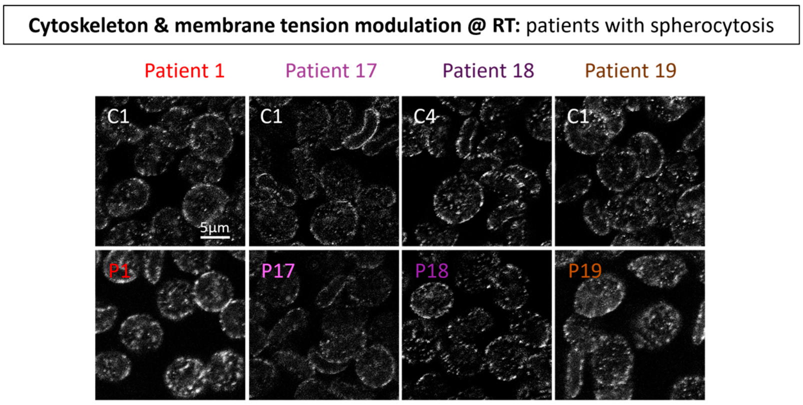

Figure S10. Alteration of Piezo1 distribution in patients with hereditary spherocytosis. RBCs from patients with hereditary spherocytosis were compared to sex-matched control (C1, male & C4, woman). Patient P1, red; P17, pink; P18, purple; P19, brown. RBCs were spread on PLL, fixed, permeabilized and immunolabeled for Piezo1. Representative imaging.

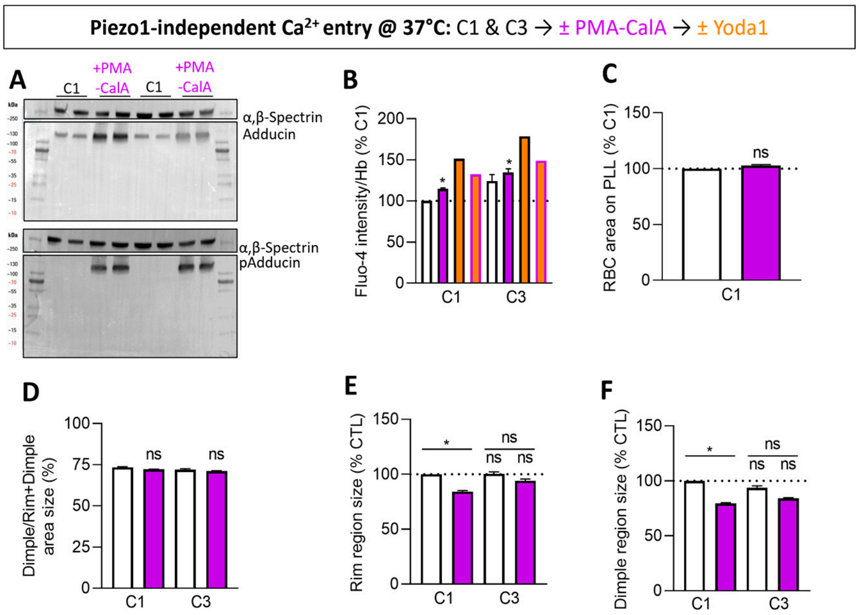

Figure S11. Cytoskeleton modulation by protein kinase C activation impairs the Yoda1-induced calcium influx but does affect neither the RBC total area nor the relative proportion of the rim and the dimple region areas. RBCs from two healthy donors (C1 & C3) were incubated at 37°C with the PMA-CalA combination for 20min in suspension (white, controls; purple, PMA-CalA-treated RBCs). After cytoskeleton modulation, RBCs were analyzed for total and phospho-α-adducin (A), intracellular Ca^{2+} levels (B), RBC projected area on PLL (C), proportion of the dimple region area over the rim one (D) and size of the local rim region (E) and dimple region (F) area. **(A)** Western Blotting images; α-β-spectrin used as control of charges. **(B)** Intracellular Ca^{2+} levels determined by fluorimetry and normalized to Hb content. Orange, 30sec Yoda1 treatment of RBCs incubated or not (black edge) with PMA-CalA (purple edge). Mean ± SEM of 3-4 independent experiments (except for the combination with Yoda1, one experiment). **(C-F)** Area size determination of living (D) and fixed and permeabilized (E-G) as in Figure S4E-H. Means ± SEM of 3 independent experiments where 20 RBCs per fluorescent images (C) and 23-55 RBCs (D-F) per image were analyzed.
